# Cross-Sectional Assessment of Sleep-Disordered Breathing Prevalence in Pediatric Population with Obesity

**DOI:** 10.3390/children13020212

**Published:** 2026-01-31

**Authors:** Abdullah Ahmed Alarfaj

**Affiliations:** Department of Surgery, College of Medicine, King Faisal University, Al-Ahsa 31982, Saudi Arabia; aalarfij@kfu.edu.sa

**Keywords:** childhood obesity, sleep-disordered breathing, neck circumference, pediatric, Saudi Arabia

## Abstract

**Highlights:**

**What are the main findings?**

- More than one-third of children with obesity screened positive for sleep-disordered breathing, indicating a substantial hidden burden in pediatric obesity clinics.
- Neck circumference emerged as a key anatomical predictor and a mediator linking overall obesity severity to sleep-disordered breathing risk.

**What are the implications of the main findings?**

- Simple anthropometric measures, particularly neck circumference, can enhance early identification of sleep-disordered breathing in obese children.
- Integrating routine sleep screening into pediatric obesity care may enable earlier intervention and improve long-term cardiometabolic and neurocognitive outcomes.

**Abstract:**

Background: Childhood obesity is a growing public health concern globally and is associated with a wide spectrum of comorbidities, including sleep-disordered breathing (SDB). SDB remains under-recognized in pediatric population with obesity, particularly in Middle Eastern settings, despite its significant impact on cognitive, behavioral, and metabolic health. Objectives: To assess the prevalence of SDB among children with obesity aged 6–12 years attending King Faisal University polyclinics in Saudi Arabia and to identify key demographic and anthropometric predictors, with particular emphasis on the mediating role of neck circumference. Methods: A cross-sectional study was conducted involving 130 children with obesity aged 6–12 years. Data collection included sociodemographic characteristics, anthropometric measurements (BMI percentile, neck and waist circumference), and screening for SDB using the validated Arabic version of the Pediatric Sleep Questionnaire Sleep-Related Breathing Disorder (PSQ-SRBD) scale. Logistic regression and mediation analyses were performed to examine associations and pathways between obesity parameters and SDB risk. Results: Of the 130 participants, 37.7% screened positive for SDB risk. SDB prevalence was higher among males and older children. Neck circumference and BMI percentile were independently associated with SDB risk, with neck circumference mediating the relationship between BMI and SDB. The mediation model indicated that increased BMI contributes to SDB risk both directly and indirectly through increased neck circumference. Conclusions: SDB is highly prevalent among obese Saudi children, and neck circumference is a significant mediator of risk. Incorporating SDB screening and neck circumference measurements into routine pediatric obesity care can facilitate early detection and management. These findings support the need for integrated, multidisciplinary approaches to improve pediatric health outcomes.

## 1. Introduction

Childhood obesity has emerged as one of the most significant public health challenges globally, with rising prevalence across both high- and low-income countries [1]. The World Health Organization (WHO) estimates that over 39 million children under the age of five were overweight or obese in 2022, a figure projected to increase steadily without strategic intervention [2]. The health implications of pediatric obesity extend far beyond metabolic syndromes, encompassing a wide range of physiological systems, including respiratory, cardiovascular, endocrine, and neurocognitive domains [3,4]. Among the less overt yet critically consequential complications is sleep-disordered breathing (SDB), an umbrella term describing a spectrum of respiratory abnormalities during sleep, ranging from primary snoring to obstructive sleep apnea (OSA) [5].

SDB in children has received increasing attention due to its established association with behavioral problems, impaired school performance, cardiovascular risk, and decreased quality of life [6]. While adenotonsillar hypertrophy remains a predominant risk factor in the general pediatric population, the role of obesity as a significant and independent risk factor for SDB is becoming increasingly evident [7,8]. Excess adipose tissue deposition in the neck and pharyngeal regions contributes to airway collapsibility, while visceral fat accumulation can reduce lung volumes, both of which potentiate upper airway obstruction during sleep [9]. Moreover, obesity-related systemic inflammation and insulin resistance may exacerbate the pathophysiology of SDB, establishing a bi-directional relationship wherein each condition compounds the other [10,11].

The prevalence of SDB among children with obesity is reported to be significantly higher than in their non-obese counterparts [7]. Studies estimate that while SDB affects approximately 1–5% of the general pediatric population, this figure may climb to 30–60% among children with obesity, depending on assessment methods and population characteristics [12]. Despite this elevated risk, SDB often remains underdiagnosed in pediatric obesity, particularly in primary care and community health settings where specialized screening tools such as polysomnography (PSG) are not routinely employed [13]. The insidious nature of SDB symptoms—such as habitual snoring, nocturnal awakenings, daytime sleepiness, and behavioral dysregulation—often leads to misattribution or oversight [14]. Given these challenges, there is a compelling need to conduct population-specific assessments of SDB prevalence within pediatric cohorts at high risk, especially those characterized by excess body weight [15].

Recent research has begun to highlight ethnic and regional differences in the presentation and severity of pediatric SDB. Factors such as craniofacial structure, socio-environmental determinants, and cultural attitudes toward sleep and body weight may influence both the manifestation and detection of SDB [16]. In regions where pediatric obesity is surging—such as the Middle East, parts of Asia, and Latin America—data on the co-prevalence and clinical characteristics of SDB remain sparse [17]. This gap in the literature underscores the importance of localized, context-sensitive research, particularly in underrepresented populations, to inform screening practices and health policy interventions.

In addition to prevalence estimates, understanding the clinical correlates and anthropometric predictors of SDB in children with obesity is essential for early detection and risk stratification [17]. Commonly used tools such as body mass index (BMI) may not fully capture the nuances of fat distribution associated with SDB risk, prompting interest in complementary measures such as neck circumference, waist-to-hip ratio, and body fat percentage [18]. Furthermore, emerging evidence suggests that subjective symptom scales, including the Pediatric Sleep Questionnaire (PSQ) and the Sleep-Related Breathing Disorder (SRBD) scale, may offer valuable, albeit imperfect, alternatives to PSG in resource-constrained settings [19]. These tools enable large-scale screening efforts and can identify children in need of further diagnostic evaluation.

Despite the growing body of literature, significant uncertainties remain regarding the true burden of SDB among pediatric population with obesity, particularly in non-Western countries [20]. Variations in study design, age stratification, diagnostic criteria, and assessment tools have contributed to inconsistencies in prevalence estimates and hindered the development of unified guidelines for screening and intervention [15]. Furthermore, few studies have employed cross-sectional population-based designs capable of generating representative prevalence data and informing public health strategies.

To address this critical gap, the present study aims to assess the prevalence of sleep-disordered breathing among children with obesity using a validated screening questionnaire in a cross-sectional design. By focusing on a defined pediatric population, this study seeks to (1) quantify the burden of SDB symptoms in relation to obesity severity, (2) identify potential demographic and anthropometric correlates, and (3) provide preliminary evidence to support the integration of routine SDB screening in pediatric obesity management. The findings have the potential to guide clinicians, educators, and policymakers in developing comprehensive care pathways that account for the complex interplay between sleep health and metabolic risk in children.

Given the public health significance of both pediatric obesity and SDB—and their reciprocal influence—the early identification and treatment of SDB in children with obesity represent a vital component of holistic pediatric care. The present study contributes to this growing field of inquiry by generating regionally relevant data that can inform clinical decision-making and enhance the quality of life for at-risk children.

### Aim of the Study

The primary aim of this study is to assess the prevalence of sleep-disordered breathing (SDB) among pediatric population with obesity using a validated screening tool. The study also seeks to explore the relationship between the severity of obesity and the presence of SDB symptoms, and to identify significant demographic and anthropometric predictors of SDB within this population. This cross-sectional analysis aims to generate region-specific evidence to inform early screening, diagnosis, and clinical management of SDB in children with obesity.

## 2. Materials and Methods

### 2.1. Study Design

This research employed a cross-sectional study design to assess the prevalence of sleep-disordered breathing (SDB) in obese pediatric patients. A cross-sectional approach was selected to provide a snapshot of the occurrence of SDB symptoms within a defined population at a single point in time, facilitating the evaluation of associations between obesity-related factors and SDB prevalence. This design is particularly well-suited for prevalence estimation and for exploring potential correlates of the condition without requiring longitudinal follow-up [21]. Data collection was conducted at the King Faisal University Polyclinics, Al-Ahsa, Saudi Arabia, between April 2025 and June 2025

### 2.2. Study Setting

The study was conducted at the Polyclinics of King Faisal University, located in the Eastern Province of Saudi Arabia. As part of a major academic medical center, the polyclinics provide comprehensive outpatient care and attract a diverse pediatric population. The site was strategically chosen for its accessibility to children attending routine follow-ups in pediatric endocrinology, nutrition, and respiratory clinics, many of whom are managing obesity or its associated comorbidities.

### 2.3. Sample and Sampling

A total of 130 obese pediatric patients aged between 6 and 12 years were recruited using a convenience sampling technique over a defined data collection period. Inclusion criteria required participants to have a body mass index (BMI) at or above the 95th percentile for age and sex, in accordance with the World Health Organization (WHO) growth standards. Children were excluded if they had known craniofacial anomalies, neuromuscular diseases, genetic syndromes, or a history of adenotonsillectomy, as these conditions may independently influence the risk of SDB. The selected sample size was based on previous literature estimating a high prevalence of SDB among children with obesity and aimed to ensure sufficient power to detect statistically significant associations.

**Sample size calculation:** The minimum required sample size was estimated using a single-proportion formula, n = Z^2^ × p(1 − p)/d^2^, assuming a 95% confidence level (Z = 1.96), an expected prevalence of SDB risk among children with obesity of approximately 38% based on published pediatric obese cohorts, and a desired precision (d) of 0.085. This yielded a minimum sample of approximately 125 participants; allowing for incomplete responses/non-response, the target sample was increased to ≥130 children. In addition, given the observed SDB-risk prevalence in our cohort (≈38%), the number of outcome events was sufficient to support multivariable logistic regression models with a limited set of prespecified predictors.

### 2.4. Data Collection Tools

Data were collected using structured questionnaires and standardized anthropometric measurements to evaluate the presence of sleep-disordered breathing symptoms and characterize obesity-related parameters.

- Sleep-disordered breathing (SDB) risk was screened using the Pediatric Sleep Questionnaire—Sleep-Related Breathing Disorder (PSQ-SRBD) scale, a validated parent-reported instrument comprising 22 items grouped into three symptom domains: snoring (9 items), sleepiness/tiredness (7 items), and behavior/inattention–hyperactivity (6 items). Responses are recorded as “yes” (1), “no” (0), or “don’t know” (missing), and the PSQ-SRBD score is calculated as the mean of non-missing item responses (range 0–1). A score ≥0.33 was used to indicate elevated SDB risk. The scoring system calculates the proportion of “yes” responses among all answered items; a score of 0.33 or higher is considered indicative of a high risk for SDB. The PSQ-SRBD has demonstrated excellent internal consistency (Cronbach’s alpha > 0.85) and good predictive validity against polysomnography, the gold standard for SDB diagnosis.
- For this study, the PSQ-SRBD was translated into Arabic using a forward-backward translation methodology involving two bilingual clinical psychologists and two pediatricians to ensure linguistic and conceptual equivalence. The translated version underwent pilot testing with a sample of 20 Arabic-speaking parents, after which minor wording adjustments were made for clarity. The final Arabic version demonstrated strong internal consistency (Cronbach’s alpha = 0.82) and was deemed both culturally and linguistically appropriate for the Saudi pediatric population. Content validity was reviewed and approved by a panel of experts in pediatric sleep medicine, psychology, and epidemiology.
- Anthropometric data included weight, height, neck circumference, and waist circumference, collected using calibrated equipment according to standard WHO measurement procedures. BMI was calculated and interpreted using WHO BMI-for-age percentiles. These variables were selected due to their known associations with upper airway obstruction and fat distribution patterns relevant to sleep-disordered breathing.

Additional sociodemographic data such as child’s age, sex, parental education level, and previous diagnoses of sleep-related or respiratory disorders were collected via structured interviews with parents or guardians during the clinic visit.

**Neck circumference (NC) measurement:** Neck circumference was measured using a flexible, non-stretchable measuring tape with the child standing upright, head in the neutral position, and shoulders relaxed. The tape was positioned horizontally at the level of the thyroid cartilage (mid-neck) and recorded to the nearest 0.1 cm. Each measurement was obtained twice by trained personnel, and the average of the two readings was used for analysis. This standardized approach is supported by evidence demonstrating excellent inter- and intra-rater reliability of NC measurement in school-aged children [22].

**NC threshold:** For descriptive analyses, NC was categorized using a pragmatic cut-off (>33 cm) consistent with prior pediatric obesity–SDB screening studies and to facilitate clinical interpretability; however, we acknowledge that optimal pediatric NC thresholds may vary by age, sex, and pubertal status and are not universally standardized. Evidence from obese adolescent cohorts has used NC ≥ 34 cm as a risk marker for OSAS, supporting the clinical relevance of elevated NC in this population [23].

**Neck-to-height ratio (NHR):** In addition to absolute neck circumference, neck-to-height ratio (NHR) was calculated as neck circumference (cm) divided by height (cm) to normalize neck size for body stature. NHR was explored as a potential anthropometric predictor of sleep-disordered breathing risk; however, preliminary analyses indicated that NHR did not provide additional predictive value beyond absolute neck circumference. Therefore, to avoid redundancy and maintain model parsimony, NHR was not included in the final regression or mediation analyses.

### 2.5. Data Collection Procedure

After obtaining ethical approval and parental consent, data collection was conducted at the polyclinics of King Faisal University by trained research assistants and clinical staff. Upon arrival, each participant’s anthropometric data were measured in a private room to ensure accuracy and confidentiality. Parents or guardians were then invited to complete the Arabic version of the PSQ-SRBD, with research staff available to assist in clarifying any items if needed. Each session lasted approximately 20–30 min. Data were reviewed on-site for completeness, and missing values were minimized by real-time verification.

### 2.6. Data Analysis

Data were analyzed using IBM SPSS Statistics version 26. Descriptive statistics (means, standard deviations, frequencies, and percentages) were used to summarize participant characteristics and PSQ-SRBD scores. The prevalence of SDB risk was estimated using the PSQ-SRBD cutoff of 0.33. Bivariate analyses, including chi-square tests for categorical variables and independent *t*-tests for continuous variables, were employed to assess associations between SDB risk and demographic or anthropometric characteristics. Furthermore, binary logistic regression was conducted to identify predictors of SDB, with results presented as odds ratios (ORs) and 95% confidence intervals (CIs). A *p*-value of <0.05 was considered statistically significant.

### 2.7. Ethical Considerations

This study was conducted in accordance with the ethical principles outlined in the Declaration of Helsinki. Ethical approval was obtained from the Institutional Review Board of King Faisal University. Written informed consent was secured from all parents or legal guardians prior to participation. For children aged 7 and above, verbal assent was also obtained in a child-friendly manner. Participant confidentiality was maintained by anonymizing data and securely storing all records. Participation was entirely voluntary, and families were informed that they could withdraw at any time without consequence to their clinical care.

**Potential sources of bias and mitigation:** Several potential sources of bias were considered. Selection bias may occur because participants were recruited from a single university polyclinic setting and participation depended on clinic attendance and caregiver consent; to reduce this risk, we applied consistent eligibility criteria and used consecutive recruitment during the study period. Information (reporting) bias is possible because SDB risk was assessed using a caregiver-reported screening questionnaire rather than polysomnography; to minimize misunderstanding, caregivers completed the questionnaire with trained staff available for clarification, and questionnaires were checked for completeness at the time of completion. Measurement bias was minimized through standardized anthropometric measurement procedures and calibrated equipment. We also limited confounding by prespecifying clinically relevant predictors (e.g., age, BMI, neck circumference) and including them in multivariable models.

## 3. Results

### 3.1. Participant Characteristics

Table 1 summarizes the demographic and clinical characteristics of the 130 obese pediatric participants. The mean age was 8.7 ± 1.8 years, with a near-equal distribution of boys (n = 66) and girls (n = 64). The majority of participants were Saudi nationals (n = 112), with the remainder being non-Saudi residents. Most parents reported a university-level education (n = 73). The mean BMI percentile was 97.4 ± 1.3.

### 3.2. Prevalence of Sleep-Disordered Breathing Symptoms

As illustrated in Table 2, 49 participants (37.7%) were classified as high risk for sleep-disordered breathing (SDB) based on the PSQ-SRBD cutoff score ≥0.33. A notably higher prevalence of SDB risk was observed among males (n = 28, 42.4%) compared to females (n = 21, 32.8%). The distribution of SDB risk by age group also demonstrated a modest increase among children aged 10–12 years

Table 3 presents the anthropometric profiles of children with and without SDB risk. Participants at risk for SDB showed higher mean BMI percentiles, neck circumferences, and waist circumferences compared to those not at risk, with statistically significant differences (*p* < 0.021 for BMI percentile; *p* < 0.007 for neck circumference). Although neck-to-height ratio (NHR) was calculated, it was not retained in the final analyses because it did not demonstrate additional discriminatory value compared with absolute neck circumference.

### 3.3. Distribution of SDB Questionnaire Scores

In Table 4, the distribution of PSQ-SRBD scores is shown across the sample. The mean total score was 0.41 ± 0.15, with the highest domain scores observed in the snoring and daytime sleepiness domains. These findings suggest that snoring and daytime sleepiness are the most prominent SDB symptoms among children with obesity in this setting.

### 3.4. Association of Demographic and Anthropometric Factors with SDB Risk

As shown in Table 5, bivariate analysis revealed significant associations between SDB risk and male gender (χ^2^ = 3.82, *p* = 0.049), age 11–12 years (χ^2^ = 4.41, *p* = 0.036), neck circumference >33 cm (χ^2^ = 5.79, *p* = 0.016), and BMI percentile ≥98 (χ^2^ = 4.27, *p* = 0.039).

### 3.5. Multivariable Logistic Regression Analysis

In Table 6, the multivariable logistic regression model shows that neck circumference and BMI percentile remained significant independent predictors of SDB risk after adjusting for age and gender. Children with a neck circumference greater than 33 cm had more than twice the odds of SDB risk (OR: 2.19; 95% CI: 1.02–4.66; *p* = 0.044). BMI percentile was also a significant predictor (OR: 1.37 per unit increase; 95% CI: 1.01–1.91; *p* = 0.038).

**Figure 1:**
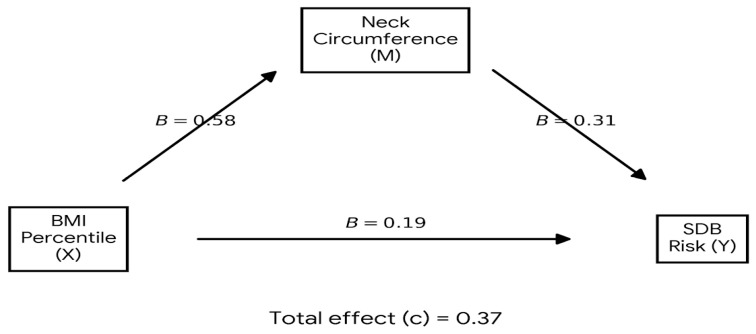
Mediation model illustrating the direct and indirect pathways linking BMI percentile, neck circumference, and risk of sleep-disordered breathing (SDB) in children with obesity. Regression coefficients (B) represent unstandardized effects.

The mediation diagram illustrates the hypothesized and empirically supported pathways linking BMI percentile, neck circumference, and the risk of sleep-disordered breathing (SDB) in children with obesity (Figure 1). As depicted, BMI percentile exerts both a direct effect on SDB risk (B = 0.19) and an indirect effect mediated through neck circumference. The positive association between BMI percentile and neck circumference (B = 0.58) highlights how increased adiposity contributes to upper airway anatomical changes. In turn, larger neck circumference is independently associated with greater risk of SDB (B = 0.31), supporting its role as a key anatomical mediator in the obesity–SDB relationship. The total effect of BMI percentile on SDB risk, calculated at B = 0.37, reflects the cumulative impact of both direct and mediated pathways. This model underscores the importance of considering both overall and regionally distributed adiposity—particularly neck circumference—when evaluating SDB risk in pediatric population with obesity. These findings highlight a clinically meaningful pathway that may inform targeted screening and intervention strategies within high-risk groups.

## 4. Discussion

This study provides important insight into the prevalence and correlates of sleep-disordered breathing (SDB) among children with obesity aged 6–12 years in a Saudi clinical setting. Our findings indicate that over a third of participants screened positive for SDB risk using the validated Arabic PSQ-SRBD scale, with higher rates observed among older children, males, and those with greater neck circumference and BMI percentiles. These results align with, and extend, previous research emphasizing the complex interplay between pediatric obesity and SDB risk factors.

The observed SDB prevalence of 37.7% is consistent with the upper range of estimates reported in both Western and Middle Eastern studies involving children with obesity [24]. For instance, Redline et al. noted that the prevalence of SDB in children with obesity can reach as high as 60%, particularly when utilizing sensitive screening instruments [25]. Similarly, a large-scale study by Verhulst et al. reported SDB rates of approximately 30–40% in obese European children, confirming the clinical significance of our findings [1]. The elevated prevalence in our sample underscores the urgent need for routine SDB screening in pediatric obesity clinics in Saudi Arabia and the broader Gulf region, where childhood obesity rates continue to rise [26].

Our analysis also highlights important demographic and anthropometric predictors of SDB risk. Male gender was associated with higher SDB prevalence, in line with established literature suggesting that boys may have a greater propensity for upper airway collapsibility and greater fat deposition in critical anatomical regions [27]. Age also emerged as a significant factor, with older children (11–12 years) displaying the highest SDB risk. This trend likely reflects the cumulative impact of prolonged obesity and pubertal changes on airway dynamics, as described in prior longitudinal analyses [28].

Anthropometric measurements, particularly neck circumference, were robustly associated with SDB risk, both independently and as a mediator between BMI percentile and SDB. Previous studies have identified neck circumference as a practical, non-invasive marker for predicting SDB in both adults and children [29]. The mediation analysis in our study supports the hypothesis that regional adiposity in the neck plays a critical mechanistic role, possibly due to increased pharyngeal tissue mass and decreased airway caliber [30]. Our findings support neck circumference (NC) as a clinically useful anthropometric marker associated with increased SDB risk among children with obesity. This is consistent with prior pediatric studies highlighting the role of upper-body adiposity in airway narrowing. Importantly, Katz et al. demonstrated that the neck-to-height ratio (NHR) may provide stronger discrimination than NC alone by accounting for somatic growth, suggesting that normalized measures may be particularly valuable in pediatric populations. While NHR was not examined in the present analysis, our results align with the broader evidence supporting NC-based screening and underscore the need for future studies to compare absolute NC and ratio-based indices across age and sex groups [31].

The direct effect of BMI percentile on SDB risk observed in our regression models corroborates the established association between obesity and sleep-disordered breathing [32]. Children with obesity are more likely to experience reduced lung volumes, elevated upper airway resistance, and chronic low-grade inflammation, all of which contribute to the pathophysiology of SDB [33]. The total effect estimated in this study (B = 0.37) closely matches the magnitude reported in a recent meta-analysis of pediatric SDB predictors, reinforcing the validity of our model [34].

Our findings support NC as a practical anthropometric marker associated with higher SDB risk in children with obesity. While NC alone can be useful in clinic-based screening, emerging evidence suggests that neck-to-height ratio (NHR) may offer stronger discrimination in growing children by normalizing NC for height. In particular, Katz et al. demonstrated that NHR performed well as a predictor of OSA among children with obesity and proposed clinically relevant thresholds [35].

Notably, the mean PSQ-SRBD scores in our cohort were highest for snoring and daytime sleepiness domains, echoing findings by Chervin et al. and others who have documented these symptoms as cardinal features of pediatric SDB [36]. These symptoms are clinically relevant as they are frequently linked to neurocognitive impairments, academic difficulties, and behavioral problems, thereby impacting overall quality of life [37]. Timely recognition and intervention are therefore crucial for mitigating long-term sequelae [38,39].

Our study adds to the limited body of research on SDB in Middle Eastern pediatric populations, especially those attending university-affiliated clinics. The validation and successful application of the Arabic PSQ-SRBD tool in this context supports its broader use for community-based screening, as recommended by recent regional guidelines [36]. Furthermore, the demonstration of neck circumference as an independent and mediating risk factor supports the integration of simple anthropometric assessments into routine pediatric evaluations [40].

Despite its strengths, including the use of a validated tool and comprehensive anthropometric measurements, this study has several limitations. The cross-sectional design precludes causal inference, and the use of a convenience sample from a single clinical center may limit generalizability. Polysomnography, the gold standard for SDB diagnosis, was not used, and thus some cases may have been misclassified despite the high sensitivity of the PSQ-SRBD. Additionally, factors such as craniofacial anomalies, genetic predispositions, and environmental influences were not systematically assessed [19,41].

Future research should include longitudinal studies across multiple centers and incorporate objective sleep measures to clarify the temporal relationship between obesity, neck circumference, and SDB risk. Interventional studies examining the impact of weight reduction and targeted therapies on SDB outcomes are also warranted, as previous work has shown promising improvements in sleep quality and metabolic health following such interventions [42,43].

### 4.1. Implications of the Study

The findings from this study have several important implications for clinical practice, public health policy, and future research. First, the high prevalence of sleep-disordered breathing (SDB) among children with obesity highlights the urgent need for systematic screening in pediatric obesity clinics, especially in regions such as Saudi Arabia where childhood obesity is rapidly increasing. Routine use of culturally adapted screening tools—such as the validated Arabic PSQ-SRBD—can facilitate early identification of children at risk, enabling timely referral for diagnostic evaluation and intervention. The identification of neck circumference as a significant mediator suggests that simple, non-invasive anthropometric measures should be incorporated into standard pediatric assessments to improve the detection of children at high risk for SDB. On a broader scale, the study underscores the importance of interdisciplinary collaboration among pediatricians, sleep specialists, nutritionists, and public health professionals to address the interconnected challenges of obesity and sleep health in children. At the policy level, these results advocate for the inclusion of sleep health promotion and SDB risk screening in school-based health programs and national childhood obesity prevention strategies. Lastly, the use of validated, locally appropriate tools provides a framework for further research and public health surveillance in the region.

### 4.2. Limitations of the Study

Several limitations should be considered when interpreting the results of this study. the cross-sectional design precludes causal inference. Second, recruitment from a single polyclinic may limit generalizability and introduces potential selection bias. While the PSQ-SRBD is a well-validated screening instrument, it is not a substitute for objective diagnostic methods such as overnight polysomnography, and thus some cases of SDB may have been misclassified. Additionally, the study did not account for other potential contributors to SDB, such as craniofacial morphology, allergic conditions, or environmental factors like household smoking. The reliance on parent-reported measures introduces the possibility of recall bias. Finally, the study did not assess longitudinal outcomes or the effects of interventions, which would provide more comprehensive insight into the natural history and management of SDB in children with obesity. This study did not include a standardized oropharyngeal examination or grading of tonsillar size/adenotonsillar hypertrophy, which is a well-recognized contributor to pediatric SDB, including among children with obesity. Therefore, we could not evaluate the independent or interacting effects of adenotonsillar hypertrophy alongside anthropometric predictors, and residual confounding is possible. Future studies should incorporate upper-airway clinical assessment and/or ENT evaluation to improve phenotyping and guide management decisions.

The age range (6–12 years) includes children who may be entering puberty; however, pubertal status (e.g., Tanner staging) was not assessed. Puberty-related hormonal and body-composition changes may influence anthropometric measures (including fat distribution and neck adiposity) and could therefore affect the observed associations with SDB risk. Future studies should incorporate Tanner staging or other markers of pubertal development to enable stratified analyses and improve the precision of anthropometric screening thresholds across developmental stages.

## 5. Conclusions

This study demonstrates a substantial burden of sleep-disordered breathing symptoms among children with obesity aged 6–12 years attending polyclinics at King Faisal University in Saudi Arabia. The findings confirm that both overall obesity and neck circumference are important predictors of SDB risk, with neck circumference mediating the relationship between BMI and SDB. These results reinforce the need for integrated, multi-disciplinary screening and intervention strategies targeting both obesity and sleep health in pediatric populations. Early identification and management of SDB may improve quality of life and long-term health outcomes for at-risk children. Future studies should expand on these findings using objective diagnostic tools, broader and more diverse populations, and longitudinal designs to inform effective prevention and treatment strategies at the population level.

## Figures and Tables

**Table 1:** Demographic and Clinical Characteristics of the Study Population (N = 130).

Variable	Value
Age, mean ± SD (years)	8.7 ± 1.8
Gender, n (%)	
Male	66 (50.8)
Female	64 (49.2)
Nationality, n (%)	
Saudi	112 (86.2)
Non-Saudi	18 (13.8)
Parental Education, n (%)	
Primary	22 (16.9)
Secondary	35 (26.9)
University	73 (56.2)
BMI percentile, mean ± SD	97.4 ± 1.3
Neck circumference (cm), mean ± SD	32.3 ± 2.9
Waist circumference (cm), mean ± SD	77.9 ± 8.1

**Table 2:** Prevalence of SDB Risk by Gender and Age Group.

Variable	N at Risk (%)	N Not at Risk (%)	Total
Gender			
Male (n = 66)	28 (42.4)	38 (57.6)	66
Female (n = 64)	21 (32.8)	43 (67.2)	64
Age Group (years)			
6–8 (n = 43)	13 (30.2)	30 (69.8)	43
9–10 (n = 54)	20 (37.0)	34 (63.0)	54
11–12 (n = 33)	16 (48.5)	17 (51.5)	33

Anthropometric Variables and SDB Risk.

**Table 3:** Anthropometric Measures by SDB Risk Status.

Measure	At Risk (n = 49)	Not at Risk (n = 81)	*p*-Value
BMI percentile	97.9 ± 1.1	97.1 ± 1.5	0.021
Neck circumference (cm)	33.4 ± 2.7	31.6 ± 2.8	0.007
Waist circumference (cm)	80.2 ± 7.8	76.4 ± 7.9	0.032
Weight (kg)	53.2 ± 8.6	47.1 ± 8.1	0.011

**Table 4:** Distribution of PSQ-SRBD Scores and Domains.

PSQ-SRBD Domain	Number of Items	Mean ± SD	Range
Total PSQ-SRBD Score	22	0.41 ± 0.15	0.14–0.81
Snoring Subscale	9	0.54 ± 0.18	0.11–1.00
Sleepiness/Tiredness Subscale	7	0.46 ± 0.20	0.00–0.86
Behavior/Inattention–Hyperactivity Subscale	6	0.33 ± 0.16	0.00–0.83

**Table 5:** Associations of Selected Variables with SDB Risk (Bivariate Analysis).

Variable	SDB Risk (%)	No SDB Risk (%)	χ^2^	*p*-Value
Male Gender	42.4	57.6	3.82	0.049
Age 11–12 years	48.5	51.5	4.41	0.036
Neck circumference > 33 cm	51.6	32.2	5.79	0.016
BMI percentile ≥ 98	46.3	53.7	4.27	0.039

**Table 6:** Multivariable Logistic Regression for Predictors of SDB Risk.

Predictor	Adjusted OR	95% CI	*p*-Value
Age (per year increase)	1.14	0.97–1.34	0.097
Male Gender	1.32	0.67–2.63	0.427
BMI percentile	1.37	1.01–1.91	0.038
Neck circumference > 33 cm	2.19	1.02–4.66	0.044

## Data Availability

Data available upon request.

## Abbreviations

BMI	Body Mass Index
C	Confidence Interval
NC	Neck Circumference
OSA	Obstructive Sleep Apnea
PSG	Polysomnography
PSQ	Pediatric Sleep Questionnaire
PSQ-SRBD	Pediatric Sleep Questionnaire–Sleep-Related Breathing Disorder scale
SDB	Sleep-Disordered Breathing
WHO	World Health Organization
